# Synthesis, Characterization, and Crystal Structure of a Diorganotin(IV) Complex with 2-Oxo-2-Phenylacetic Acid 4-Hydroxybenzohydrazone

**DOI:** 10.5402/2011/708162

**Published:** 2011-04-11

**Authors:** Jing Li, Handong Yin, Min Hong

**Affiliations:** Department of Chemistry, Liaocheng University, Liaocheng 252059, China

## Abstract

The complex dibutyltin 2-oxo-2-phenylacetic acid 4-hydroxybenzohydrazone has been synthesized and characterized by elemental analysis, IR, ^1^H and ^13^C NMR, and X-ray single-crystal diffraction studies. The crystal structure belongs to triclinic, space group P-1 with *a* = 9.3220 (10) Å, *b* = 9.8779 (11) Å, *c* = 15.9401 (17) Å, *β* = 97.0930 (10)°, *Z* = 2, *V* = 1427.6(3) Å^3^, *Dc* = 1.413 mg/cm^3^, *μ* = 0.936 mm^−1^, *F*(000) = 628, *R* = 0.1158, and *wR* = 0.2522. X-ray analysis indicates that O(2), N(2), O(4), and O(4)#1 from the ligand and O(5) from ethanol molecule are in the equatorial positions; the axial positions are occupied by two *n*-butyl groups. It shows a distorted pentagonal bipyramid configuration with seven-coordination for central tin atom. Fascinatingly, the supramolecular infrastructures are observed, which exist as two-dimensional sheets assembled from the organometallic subunits through intermolecular and intramolecular O–H*⋯*X or C–H*⋯*X (X = O or N) hydrogen bonds.

## 1. Introduction

Organotin complexes have attracted more and more attention in recent years, partly owing to their determinately or potentially pharmic value, which have been reported many times before [1], and also for the versatile molecular structure and supramolecular architecture exhibited by these complexes [2–4]. Among these, Schiff base organotin complexes have received much attention, as such ligands and their metal complexes are reported to exhibit bactericidal and antitumor activities [5, 6]. The coordination chemistry of tin is extensive with various geometries and coordination numbers known for both inorganic and organometallic complexes [7]. Especially, organotin complexes react with the ligands with nitrogen atoms, yielding products characterized by Sn–N bonds [8]. Studies of Schiff base organotin complexes containing carboxylate ligands with additional donor atoms (e.g., N, O or S), which are available for coordinating to tin atom, have revealed that new structural types may lead to different activities [9]. In our previous work, we have reported several novel molecular structures of organotin complexes with Schiff bases containing carboxylate [10–14]. As an extension of studies of organotin complexes with Schiff base, we synthesized a new diorganotin complex of 2-oxo-2-phenylacetic acid 4-hydroxybenzohydrazone by the reaction of di-*n*-butyltin dichloride and ligand in 1 : 1 stoichiometry. The synthesis, structure, and spectra characterizations of the complex are reported herein. X-ray diffraction shows that the central tin atom is seven-coordinate by the two *n*-butyl carbon atoms, the imino nitrogen atom, and four oxygen atoms from ethanol and the ligand. The supramolecular infrastructures are observed, which exist as two-dimensional sheets assembled from the organometallic subunits through intermolecular and intramolecular O–H*⋯*X or C–H*⋯*X (X = O or N) hydrogen bonds. 

## 2. Experimental

### 2.1. Materials and Physical Measurements

Di-*n*-butyltin chloride, 4-hydroxybenzohydrazide, and 2-oxo-2-phenylacetic acid were commercially available and used without further purification. All the solvents used in the reaction were of AR grade and dried using standard literature procedures. The melting points were obtained with Kofler micromelting point apparatus and were uncorrected. IR spectra were recorded with a Nicolet-460 spectrophotometer using KBr discs and sodium chloride optics. ^1^H and ^13^C NMR spectra were recorded with a Varian Mercury Plus 400 spectrometer. The chemical shifts are reported in ppm with respect to the references and are stated relative to the inner reference tetramethylsilane for ^1^H, ^13^C NMR spectroscopy. Elemental analyses were performed with a PE-2400 II elemental analyzer. 

### 2.2. Synthesis of Schiff Base Ligand 1 (2Z)-[(4-Hydroxybenzoyl) Hydrazono] (Phenyl) Acetic Acid

The Schiff base ligand 1 was prepared from the benzoylformic acid and 4-hydroxybenzoic hydrazide in ethanol solution. A mount of yellow power was obtained. Yield: 85%. M.p.: 199–201°C. Anal. Calc. for C_15_H_12_N_2_O_4_: C, 63.38; H, 4.25, N, 9.85%. Found: C, 63.30; H, 4.22; N, 9.79%.

### 2.3. Preparation of the Complex 1

The reaction was carried out under nitrogen atmosphere with the use of standard Schlenk technique. The ligand 1 (0.28 g, 1.0 mmol) and sodium ethoxide (0.07 g, 1.0 mmol) were added to a solution of absolute ethanol (30 mL) and heated under reflux with stiring for 0.5 h. After the addition of di-*n*-butyltin chloride (0.30 g, 1.0 mmol) to the reactor, the reaction mixture was refluxed for 10 h more. The reaction solution thus obtained was filtered and evaporated under vacuum to form a yellow solid and then recrystallized from dichloromethane/hexane to give yellow single crystal of complex. Yield: 70%. M.p.: 168–170°C. Anal. Calc. for C_27_H_40_N_2_O_6_Sn: C 53.40; H 6.64; N 4.61%. Found: C 53.35; H 6.29; N 4.58%. IR (KBr, cm^−1^): 1608, 1369 (m, CO_2_), 1635 (s, C=N), 1608 (m, C=N–N=C), 570 (m, Sn–O), 537 (w, Sn–C). ^1^H NMR (400 MHz, CDCl_3_, 298 K, ppm): 0.96 (t, 6H, CH_3_), 1.37–1.42 (m, 8H, –CH_2_CH_2_–), 1.68–1.82 (m, 4H, Sn–CH_2_-), 1.42 (t, 3H, –CH_2_–CH_3_), 3.67 (m, 2H, –O–CH_2_–), 8.53 (s, 1H, –OH), 3.57 (m, 2H, –O–CH_2_–), 1.11 (t, 3H, –CH_3_), 2.10 (s, 1H, –OH), 7.26–7.59 (m, 5H, –C_6_H_5_), 6.86–7.49 (m, 4H, –C_6_H_4_–O–), 5.40 (s, 1H, Ph–OH). ^13^C NMR (100 MHz, CDCl_3_, 298 K, ppm): 173.68, 169.53, 160.78, 142.85, 133.24, 131.12, 130.67, 129.25, 128.94, 122.56, 116.02, 57.94, 57.93, 27.06, 21.93, 21.42, 17.45, 17.42, 13.81.

### 2.4. Structure Determination

**Figure 1:**
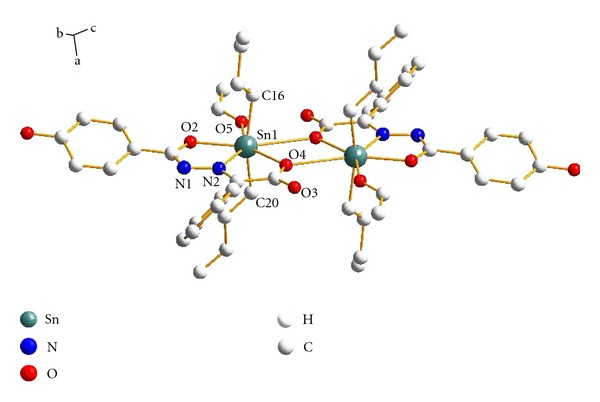
Molecular structure of the complex.

**Figure 2:**
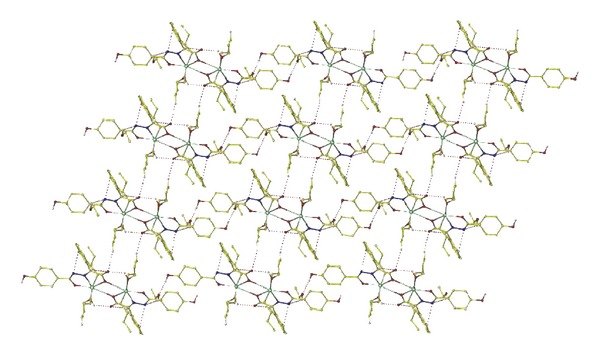
Two-dimensional sheets, formed by O–H*⋯*X, C–H*⋯*N (X = O,N) hydrogen bonds.

A yellow single crystal with the dimensions of 0.50 mm × 0.46 mm × 0.41 mm was mounted on a Bruker SMART CCD 1000 diffractometer equipped with a graphite-monochromatic Mo-K*α* (*λ* = 0.71073 Å) radiation by using a **ω**scan mode [2.21 < *θ* < 25.02] at 298(2) K. A total of 7006 reflections were collected with 4855 unique ones (Rint = 0.0887), of which 330 were observed with I > 2*σ*(I) and used in the succeeding refinements. Intensity data were corrected for Lp factors and empirical absorption. The structure was solved by direct methods and expanded by difference Fourier techniques with SHELXS-97 program [15, 16]. All of the nonhydrogen atoms were located with successive difference Fourier syntheses. The structure was refined by full-matrix least-squares techniques on *F* ^2^ using anisotropic thermal parameters for all nonhydrogen atoms. The hydrogen atoms were added according to theoretical models. The final full-matrix least-squares refinement converged at *R* = 0.1158 and *ωR* = 0.2522  (*ω* = 1/[*σ* ^2^(*F*_*o*_^2^) + (0.1586*P*)^2^], where *P* = (*F*_*o*_^2^ + 2*F*_*c*_^2^)/3), *S* = 1.03, (Δ*ρ*)_max⁡_ = 1.45, (Δ*ρ*)_min⁡_ = −1.92e/Å^3^, and (Δ/*σ*)_max⁡_ = 0.007. The molecular structure and two-dimensional sheets of the title complex are shown in Figures 1 and 2, respectively. The crystal data and structure refinement are listed in Table 1. The selected bond lengths and bond angles are listed in Table 2. The hydrogen bond lengths and bond angles are listed in Table 3. 

## 3. Results and Discussion

### 3.1. Synthesis of Organotin(IV) Complex 1

See Scheme 1.

### 3.2. Spectral Characterization

The IR spectra show the metal-ligand bonds formation through –CO_2_^−^, –O^−^, and –N sites, and the associated Sn–O–Sn, Sn–O, and Sn–N absorption values also support this. The absence of the N–H and C=O stretching vibration bands is consistent with the deprotonation of the CO–NH groups and coordination to the organotin(IV) ions in the enol form. And the characteristic absorption at 1608 cm^−1^ indicates the presence of C=N–N=C group [17], thus indicating the ligand coordinate to the tin centre in an enolic form, which is in accordance with the X-ray structure analysis and their corresponding reaction mechanism. The ^1^H NMR spectrum of the complex shows that those signals of –OH and –CONH protons in the spectrum of the ligand are absent, thus indicating the removal of those protons and the formation of Sn–O bonds. The ^13^C NMR spectrum of the complex shows that the chemical shift of the carboxylate carbon shifts to a lower field region in almost all the organotin(IV) derivatives indicating participation of the carboxyl (COO) group in coordination to tin atom [18]. On the basis, we can conclude that the tin atom of complex in solution is seven-coodinated, and this can be confirmed by the X-ray crystal structures. 

### 3.3. Description of the Crystal Structure

The crystal and unit cell structures are given in Figures 1 and 2, respectively. As illustrated in Figure 1, the environment of Sn(1) is in a distorted pentagon bipyramid with a nitrogen atom, four oxygen atoms, and two *n*-butyl groups (C(20)–Sn(1)–C(16) = 161.8(7)°, which is deviated from linear angle 180°). It is a centrosymmetric arrangement leading to a Sn_2_O_2_ core connected by the Sn–O bond, and the Sn(1)–O(4)#1 bond distance is 2.805(11) Å, is greater than the sum of the covalent radii of Sn and O (2.56 Å), but is considerably less than the sum of the Van der Waals radii Sn–O (3.68 Å), indicating the weak bonding interaction between Sn(1) and O(1)#1. The Sn(1)–N(2) distance is 2.258(12) Å, which is greater and longer than the sum of the covalent radii of Sn and N (2.15 Å) but is considerably less than the sum of the Van der Waals radii (3.75 Å) and should be considered as bonding interactions [19]. It is noteworthy that the solvent molecule-ethanol involved in the coordination. Fascinatingly, the supramolecular infrastructures were observed in the complex, which exist as two-dimensional sheets assembled from the organometallic subunits through intermolecular and intramolecular O–H*⋯*X or C–H*⋯*X (X = O or N) hydrogen bonds. 

## Figures and Tables

**Scheme 1:**
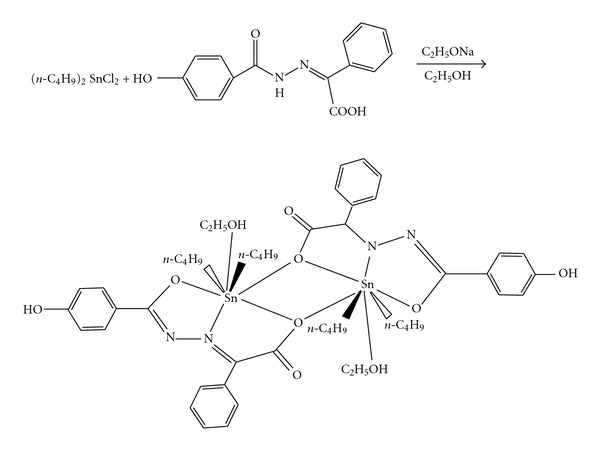
Synthesis of organotin(IV) complex 1.

**Table 1:** Crystal data and structure refinement for complex 1.

Complex	1
Empirical formula	C_27_H_40_N_2_O_6_Sn
Formula weight	607.30
Temperature (K)	298(2)
Crystal system	Triclinic
Space group	P-1

Unit cell dimensions	

*a* (Å)	9.3220(10)
*b* (Å)	9.8779(11)
*c* (Å)	15.9401(17)
*α* (°)	100.622(2)
*β* (°)	97.0930(10)
*γ* (°)	92.8550(10)
Volume (Å^3^)	1427.6(3)
Z	2
Calculated *d* (g/cm^3^)	1.413
Absorption coefficient (mm^−1^)	0.936
*F* (000)	628
Crystal size (mm)	0.50 × 0.46 × 0.41
Theta range for data collection (°)	2.21–25.02
Limiting indices	−11 ≤ *h* ≤ 11
	−11 ≤ *k* ≤ 9
	−15 ≤ *l* ≤ 18
Reflections collected/unique	7006/4855 [*R*(int) = 0.0887]
Completeness to theta = 25.02°	96.3%
Absorption correction	Semi-empirical from equivalents
Max. and min. transmission	0.7002 and 0.6519
Refinement method	Full-matrix least-squares on *F^2^*
Data/restraints/parameters	4855/0/330
Goodness-of-fit on *F^2^*	1.031
Final *R* indices [I > 2*σ*(I)]	*R_1_* = 0.1158, *wR_2_* = 0.2522
*R* indices (all data)	*R_1_* = 0.2161, *wR_2_* = 0.3313
Largest diff. peak and hole (e·Å^−3^)	1.446 and −1.923

**Table 2:** Selected bond lengths (Å) and bond angles (°).

Sn(1)–C(20)	2.041(19)	Sn(1)–C(16)	2.085(15)
Sn(1)–O(2)	2.140(12)	Sn(1)–N(2)	2.258(12)
Sn(1)–O(4)	2.260(10)	Sn(1)–O(5)	2.398(11)
Sn(1)–O(4)#1	2.805(11)		
C(20)–Sn(1)–C(16)	161.8(7)	O(2)–Sn(1)–N(2)	69.5(4)
N(2)–Sn(1)–O(4)	69.6(4)	O(2)–Sn(1)–O(5)	79.0(4)
O(5)–Sn(1)–O(4)#1	75.2(4)	O(4)–Sn(1)–O(4)#1	66.7(4)

Symmetry code: #1 – *x* + 2, −*y*, −*z* + 1			

**Table 3:** Hydrogen bond lengths (Å) and bond angles (°).

D–H*⋯*A	d(D–H)	d(H*⋯*A)	d(D*⋯*A)	<(DHA)
C(10)–H(10)*⋯*N(1)	0.93	2.60	2.94(2)	101.7
C(14)–H(14)*⋯*O(3)	0.93	2.53	2.91(2)	104.4
O(5)–H(5A)*⋯*O(3)#1	0.85	2.08	2.598(16)	118.4
O(6)–H(4)*⋯*N(1)#2	0.82	2.29	2.898(18)	131.8
O(1)–H(1)*⋯*O(6)#3	0.82	1.88	2.69(2)	168.3
C(19)–H(19A)*⋯*O(3)#4	0.96	2.60	3.52(4)	161.3

Symmetry code: #1 –*x* + 2, −*y*, −*z* + 1 #2 –*x* + 1, −*y*, −*z* + 1 #3 *x*, *y*, *z*−1 #4 *x*−1, *y*, *z *				
